# Transient Central Diabetes Insipidus and Marked Hypernatremia following Cardiorespiratory Arrest

**DOI:** 10.1155/2017/1574625

**Published:** 2017-07-03

**Authors:** Sahar H. Koubar, Eliane Younes

**Affiliations:** ^1^Department of Medicine, Division of Nephrology, American University of Beirut Medical Center and School of Medicine, Beirut, Lebanon; ^2^Department of Medicine, Division of Nephrology, Sacre Coeur Hospital, Hazmieh, Lebanon

## Abstract

Central Diabetes Insipidus is often an overlooked complication of cardiopulmonary arrest and anoxic brain injury. We report a case of transient Central Diabetes Insipidus (CDI) following cardiopulmonary arrest. It developed 4 days after the arrest resulting in polyuria and marked hypernatremia of 199 mM. The latter was exacerbated by replacing the hypotonic urine by isotonic saline.

## 1. Introduction

Central Diabetes Insipidus (CDI) is characterized by the lack of antidiuretic hormone (ADH) causing loss of urinary concentrating ability, polyuria, thirst, and hypernatremia. ADH is an octapeptide synthesized in the supraoptic nuclei of the hypothalamus and then stored and released from the posterior pituitary gland. It maintains normal serum osmolality by promoting free water reabsorption in the collecting ducts of the nephron. The known causes of CDI include germinoma/craniopharyngioma; Langerhans cell histiocytosis; local inflammatory, autoimmune, or vascular diseases; trauma resulting from surgery or an accident; sarcoidosis; metastases; and midline cerebral and cranial malformations [1, 2]. CDI related to cardiorespiratory arrest and anoxic brain injury has been reported but appears to be an overlooked complication [3–7]. We describe a patient who experienced a transient form of CDI with marked hypernatremia following cardiorespiratory arrest and anoxic brain injury.

## 2. Case Presentation

A 43-year-old woman with advanced amyotrophic lateral sclerosis, maintained on home ventilator, was transferred to our institution after being resuscitated following an out-of-hospital cardiorespiratory arrest. She had initially sustained a respiratory arrest secondary to pneumonia and mucus plugging which ultimately resulted in pulseless electrical activity. She was unresponsive for 30 minutes before she received resuscitation. Elapsed time before return of spontaneous circulation was 30 minutes. Upon arrival to our institution, she was unresponsive with fixed dilated pupils and no spontaneous respiratory effort. She was also in septic shock secondary to pneumonia. She received intravenous hydration with 0.9% saline, norepinephrine infusion, and broad spectrum antibiotics (piperacillin-tazobactam and vancomycin). Upon presentation, her laboratory values showed marked leucocytosis of 37,600/cm^3^ with a neutrophilic shift. Her serum creatinine was 0.2 mg/dl, blood urea nitrogen 13 mg/dl, sodium 137 mM, potassium 3.6 mM, chloride 98 mM, bicarbonate 19 mM, and lactate 5 mM. Her PCO2 on arterial blood gases was 48 mmHg. Her serum aspartate transaminase and alanine transaminase were 307 and 177 IU/L, respectively. Her brain computed tomography was consistent with hypoxic ischemic brain injury.

**Figure 1:**
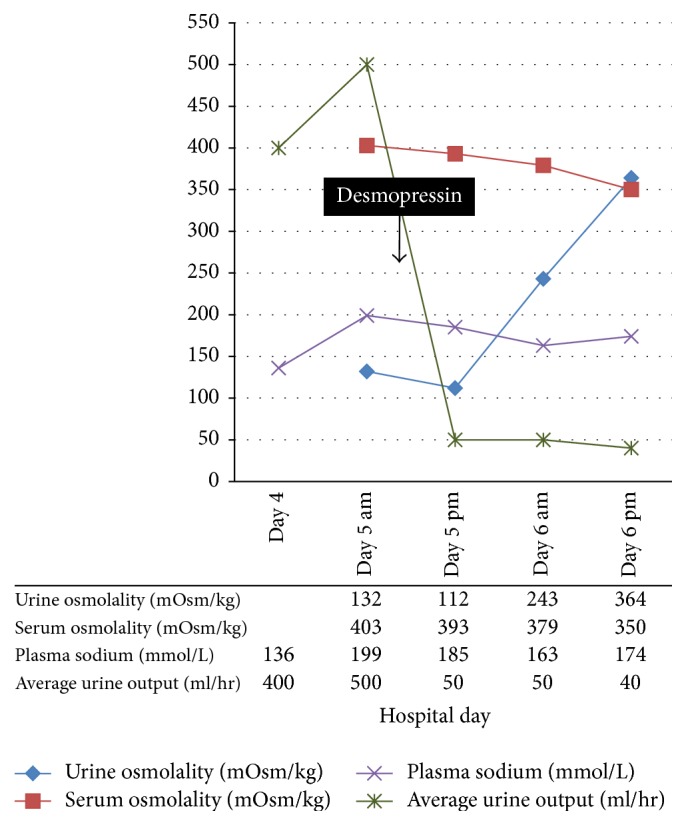
Changes in plasma sodium, plasma and urine osmolality, and urine output before and after desmopressin.

Her hemodynamic condition stabilized over the next 72 hours of hospitalization. However, on hospital day 5, the nephrology team was consulted for a significant elevation in serum sodium level from 136 to 199 mM within 24 hours. This was repeated twice with similar results and a measured plasma osmolality was 403 mOsm/kg. Her concomitant serum potassium was 2.3 mM, chloride 171 mM, and bicarbonate 15 mM. She had been polyuric for the previous 36 hours with an hourly urine output of 300–600 ml, and this has been replaced by approximately 10 liters of 0.9% NaCl (a total of 1500 mmoles of sodium) at a rate of 0.25–1 L per hour. Spot urine sodium concentration was 38 mM and urine osmolality was 112 mOsm/kg, suggestive of predominantly free water diuresis. Hence, a diagnosis of Diabetes Insipidus was made. The patient was given 1 L of 5% dextrose in water over 1 hour followed by an infusion at 225 ml/hour, together with 1 mcg of desmopressin parenterally. Following desmopressin administration, urine output dropped significantly to 50 ml/hour; urine osmolality increased to 243 mOsm/kg (in 12 hours) and to 364 (in 24 hours) (Figure 1). Her serum sodium concentration decreased to 185 mM within the following 12 hours. A diagnosis of CDI was contemplated.

On hospital day 7, the patient developed oliguric ischemic acute kidney injury (AKI) secondary to intravascular volume depletion and hypotension. Her serum creatinine increased and then plateaued at 1.1 mg/dl for 14 days and was down to 0.6 mg/dl by hospital day 30. Her sodium came down to 158 mM on day 7, 151 on day 15, and 145 on day 39. That change in sodium has been achieved by free water only. Her CDI was transient and she did not require any further doses of desmopressin. She did, however, develop central hypothyroidism and central adrenal insufficiency for which she was receiving thyroxine replacement and hydrocortisone, respectively. The patient was transferred to another hospital on day 45 of hospitalization.

## 3. Discussion

The incidence of CDI among cardiopulmonary arrest survivors varies between 12 and 21% [8, 9]. Recently, more cases have been recognized and reported. That is likely secondary to better care following cardiac arrest.

Though the brain consumes 1/5 of the body's oxygen requirements, the hypothalamus is relatively resistant to hypoxic injury given its extensive collateral blood supply [10]. ADH deficiency develops only after extensive destruction of the hypothalamic neurosecretory cells [11]. Cellular damage might be further exacerbated by the hypertonicity secondary to hypernatremia which might decrease local oxygen consumption and worsen acidosis [12]. The hypercapnia associated with cardiac arrest also contributes to neuronal injury [13]. In some cases, ADH deficiency might go unnoticed as the clinical picture is masked by oliguric AKI secondary to the hypoperfusion/shock state following cardiorespiratory arrest. Other patients might not live long enough to manifest the clinical picture of polyuria and hypertonic dehydration.

The transient nature of CDI in our patient might reflect edema or injury rather than complete destruction of the hypothalamic/hypophyseal tissue. Though the patient had hypokalemia, which can cause nephrogenic DI, her significant response to desmopressin suggests a major component of ADH deficiency and CDI. The replacement of hypotonic urine with isotonic saline further exacerbated her hypernatremia.

The development of CDI is usually an ominous prognostic sign. In a retrospective study by Chae et al. [8] and another by Lee et al. [9], people with CDI after cardiac arrest had poor neurologic outcomes. CDI was also associated with high mortality or brain death especially when it had an early onset (within 7 days).

Our case highlights the importance of monitoring for CDI following cardiac arrest. Front line physicians including emergency medicine physicians and intensivists should be aware of such complication which can be fatal if not discovered and treated early. They should watch for polyuria following cardiac arrest which can be the early sign for DI. Early treatment with desmopressin may reduce hypernatremia and perhaps secondary brain injury [12]. Also the choice of the replacement fluid could be detrimental. Hypotonic fluids should be used to replace the hypotonic urine; otherwise, fatal hypernatremia can develop. Timely management may result in better neurologic outcomes or may allow adequate time for organ procurement in brain dead patients.

## Abbreviations

mM: millimolar.
